# The lowest point of fibula (LPF) could be used as a reliable bony landmark for arthroscopic anchor placement of lateral ankle ligaments ----compared with open Broström procedure

**DOI:** 10.1186/s12891-023-06876-y

**Published:** 2023-09-26

**Authors:** Xin Xie, Linxin Chen, Cunshuai Fan, Shouyi Song, Yin Yu, Chen Jiao, Yanbin Pi

**Affiliations:** 1 Institute of Sports Medicine, Peking University Third Hospital, 49 North Garden Road, Haidian District, Beijing, 100191 PR China; 2 Orthopedics Dept.1, Pingdingshan first people’s Hospital, Pingdingshan city, Henan province PR China

**Keywords:** Lateral ankle ligament, Arthroscopic ligament repair, Fibular footprint, Arthroscopic anatomy, IV, Prospective comparative investigation

## Abstract

**Background:**

Arthroscopic technique procedures was wide accepted for the treatment of chronic ankle instability (CAI). But little acknowledge was involved to the bony landmarks and anatomic features of different bundles of lateral ligaments under arthroscopic view.

**Methods:**

Sixty patients with acute or chronic lateral ankle ligaments injury (LAI) were collected prospectively, and divided randomly into two groups. In arthroscopic group, the bone tunnels were made on the LPF arthroscopically. And in open group, the bone tunnels were made on the Fibular obscure tubercle (FOT) in open procedure. The inferior bundle of ATFL and Arcuate fibre was also identified reference to the LPF and labeled by a PDS II suture penetration. Following that, The distances of the bone tunnels to the different bony markers were measured and compare between two groups. The penetrating locations of PDS II on the inferior bundle of ATFL and Arcuate fibre were also confirmed intraoperatively. And the safe angle of anchor implantation on the axial view was measured on postoperative CT scan.

**Results:**

The distances of bone tunnel to the fibular tip, the fibular insertion of anterior-inferior tibiofibular ligament (AITFL), and the FOT in arthroscopic and open locating groups were 4.9 ± 2.2 and 6.3 ± 2.2 mm, 13.5 ± 2.7 and 12.4 ± 1.1 mm, 5.8 ± 2.2 and 5.6 ± 1.0 mm, respectively. The distances of bone tunnels to the FOT and fibular tip on 3d-CT view was 4.4 ± 1.5 and 4.6 ± 0.9 mm, 14.4 ± 3.2 and 13.2 ± 1.8 mm in arthroscopic and open group, and there were no significant differences between two groups. The safe angle of arthroscopic anchor placement on the axial plan was ranged from 24.9 ± 6.3^o^ to 58.1 ± 8.0^o^. The PDS II sutures penetrating on the inferior bundles of ATFL and the arciform fibres were also comfirmed successfully by open visualizaion.The average distance of penetration point to the horizontal line cross the fibular tip was 2.3 ± 2.7 mm (ranged from − 3.1 to 6.0 mm), and to the vertical line cross the FOT was 2.7 ± 2.7 mm (ranged from − 2.5 to 7.5 mm).

**Conclusion:**

Take the lowest point of fibula under arthroscopy (LPF) as a bony reference, we could identify the iATFL under arthroscopic visualization. By this way, we could place the suture anchors properly to the fibular footprint and suture the iATFL fibres successfully.

**Supplementary Information:**

The online version contains supplementary material available at 10.1186/s12891-023-06876-y.

## Background

About 20–40% of Lateral ankle ligaments injuires will be failed to the conservative treatment and required surgical intervention [1]. In 1966, Broström firstly described an anatomical repair technique for lateral ankle ligament injury [2]. Following that, Gould [3] and Kalsson [4], modified this surgical technique by shortening and reinsertion the ligaments on the anterior border of fibular with bone tunnals or Suture anchors fixation.

Recently, the arthroscopic technique was widely used to repair the lateral ligaments of the ankle and yielding good outcomes with fewer complications [5]. On the basis of Giza’s studies [6, 7], the biomechanical analysis was performed and compared between arthroscopic and traditional open procedure, and there was no significant difference between two procedures. Compared with open procedure, the arthroscopic treatment of chronic ankle instability was showing advantages of minimal incision, lower complication of nerve injury [8, 9].

The developments of those surgical procedures were based on the knowledge of anatomical studies. Well understanding the anatomic feature and footprints of ATFL and CFL could help us to implant the suture anchors and repair the ligaments precisely.

Several studies focused on the arthroscopic anatomy of lateral ankle ligaments [10] [11] [12]. They believed that ATFL’s superior fascicle was an intra-articular structure, and could be visualized arthroscopically. While, the inferior fascicle of ATFL was an extra-articular structure, connected with CFL by arciform fibers and shared a common fibular origin located proximal to the fibular tip and just below the fibular insertion of superior ATFL fascicle (sATFL), but it couldn’t be intraarticularly visualized under arthroscopy. In cadaveric studies, researchers provided a method to explore the iAFTL and CFL arthroscopically by dissecting the the ATFL and lateral ankle joint capsular from the fibular insertion completely [13] [14]. But the integrity of lateral ligaments and joint capsule couldn’t be maintained.

Vega J et al [15], provided a anatomic evidence of widely connection between iATFL, CFL (arciform fibres) and PTFL. They also implicated that this connected structure is very importance for arthroscopic repairing. A biomechanical study also revealed that the iATFL and CFL were connected by acurate fibers which are robust enough to transmit tension form one sturcture to the other. they concluded that the proximal lesions of the iATFL and CFL can be repaired together by one single suture [16].

Surporting by the studies mentioned above, exploration and suturing the iATFL under arthroscopy is very important for lateral ankle ligament repairing. but how to identify the anatomic feature and the fibular insertion of iATFL is a great challenge during arthroscopic exploration.

In present study, we hypothesis that the lowest point of fibula (LPF) under arthroscopy is corresponding to the Fibular obscure tubercle (FOT), and could be used as a bony marker to identify the iATFL and its insertion. The iATFL is the fibers running below this point and connecting with CFL and PTFL. Using LPF as a bony reference, we could identify the iATFL and its common insertion with CFL, it may help us to implant the suture anchors and repair the iATFL precisely and efficiantly.

## Materials and methods

### Patient characteristics and inclusion/exclusion criteria

**Fig. 1:**
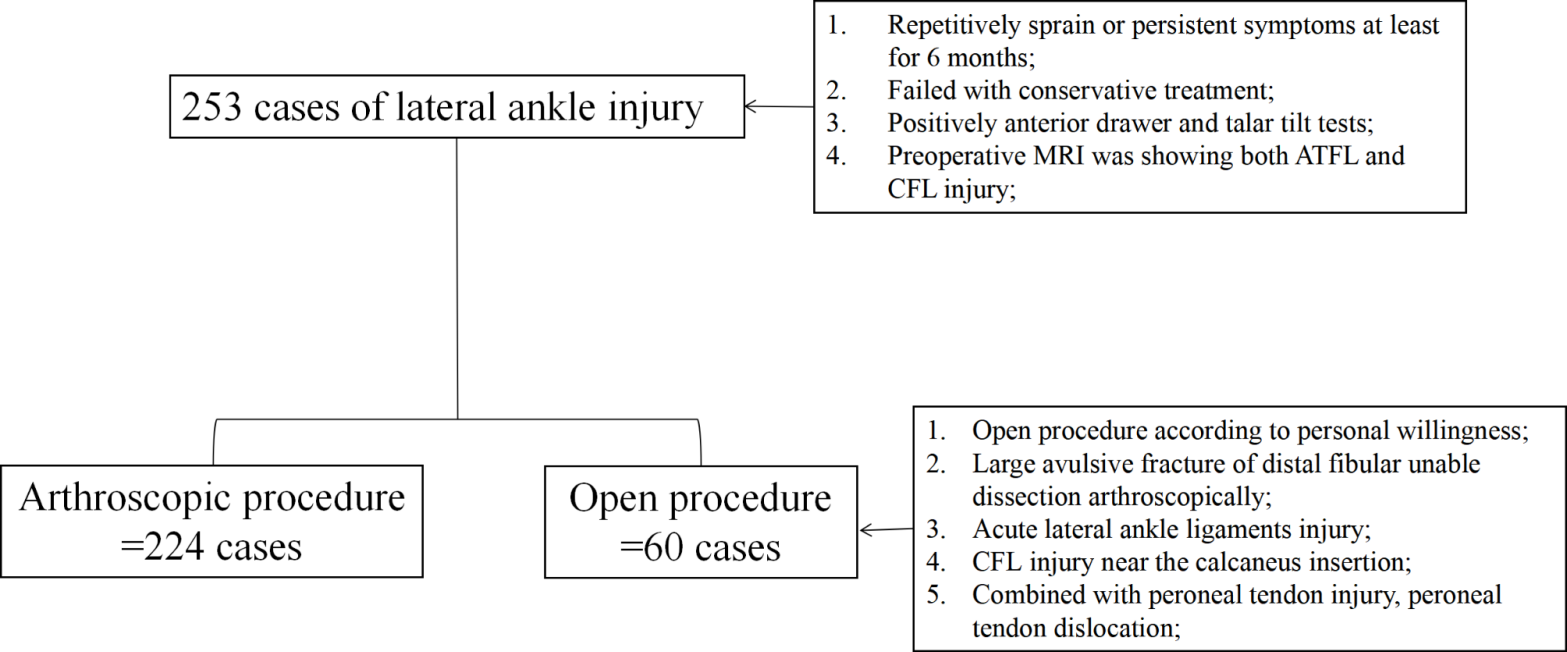
Enrollment flowchart

Between April 2021 to July 2021, 253 cases of lateral ankle injury who were planned to be treated surgically at our hospital were collected prospectively according to the including and excluding criteria. And all patients were treated surgically by three surgeons specializing in foot and ankle sports medicine. Among those cases, 60 patients were required to perform traditional open insertional repairing procedure because of the patient’s personal willingness or the reasons unsuitable of arthroscopic repair (Fig. 1).

The clinical inclusion criteria were included: lateral ankle ligaments injury (LAI) with repetitively sprain or persistent symptoms of pain and swelling and failed with conservative treatment; acute lateral ankle ligaments injury; Positively anterior drawer and talar tilt tests; Preoperative MRI was showing both ATFL and CFL injury; the conditions requiring open procedures: large avulsive fracture of distal fibular unable dissection arthroscopically, CFL injury near the calcaneus insertion, combined with peroneal tendon injury, peroneal tendon dislocation. Exclusion criteria were included: Previous ankle surgeries; MRI were showing absence of ATFL or CFL which was suitable for ligament reconstruction; Infection involving ipsilateral ankles, Congenital ankle deformity or disease, ankle fracture or dislocation requiring surgical intervention (including medial/lateral/posterior malleolar and talar/calcaneus fractures, not including lateral and medial malleolar avulsion fracture). Sixty patients were divided into two groups: arhroscopic locating group (29 cases) and open locating group (31 cases).

### Surgical procedures and intraoperative measurements

#### Arthroscopic locating group

**Fig. 2:**
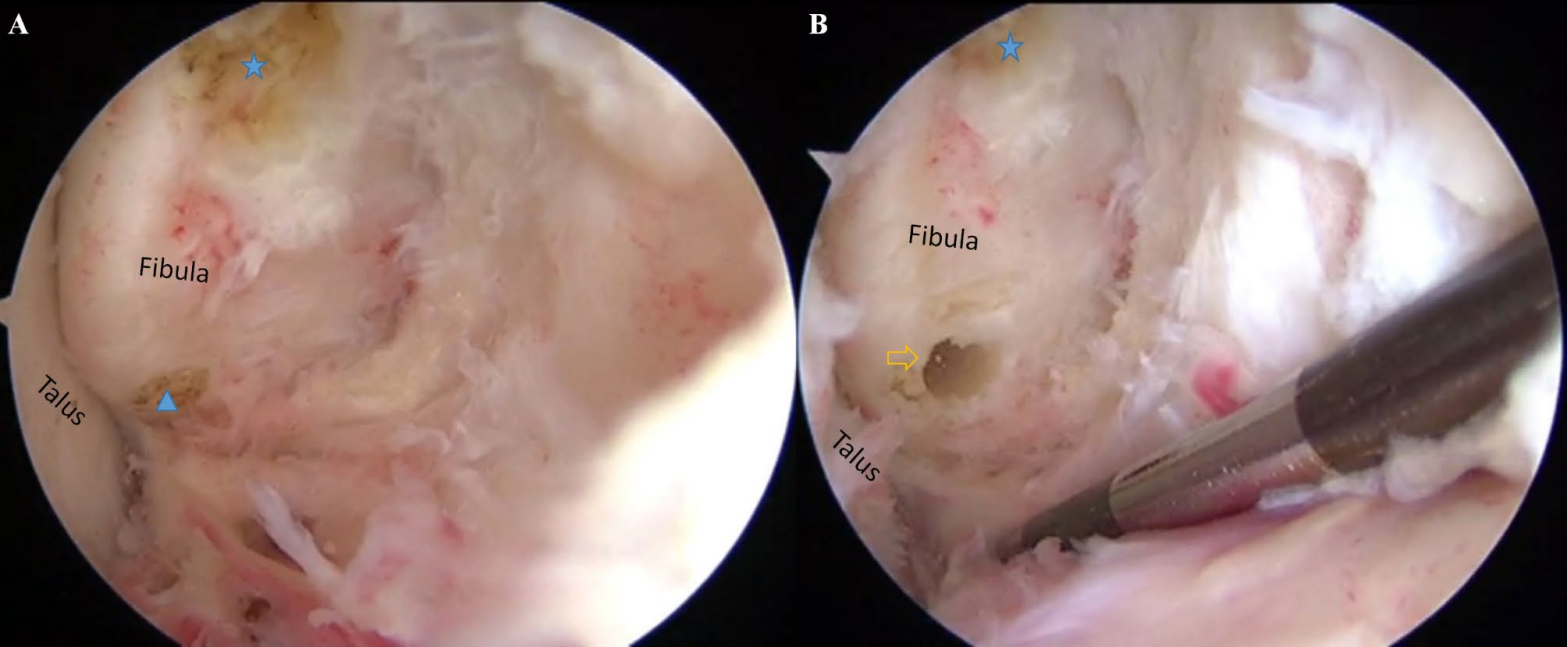
Arthroscopic view of anterior-lateral gutter of ankle joint for anterior-medial portal. The ATFL was ruptured from the fibular insertion, the lowest point of fibula (LPF) and the fibular insertion of anterior inferior tibiofibular ligament (AITFL) were identified and labeled (**A**, LPF: blue solid triangle, AITFL: blue solid star). A bone tunnel was drilled just above the LPF (**B**, empty orange arrow)

**Fig. 3:**
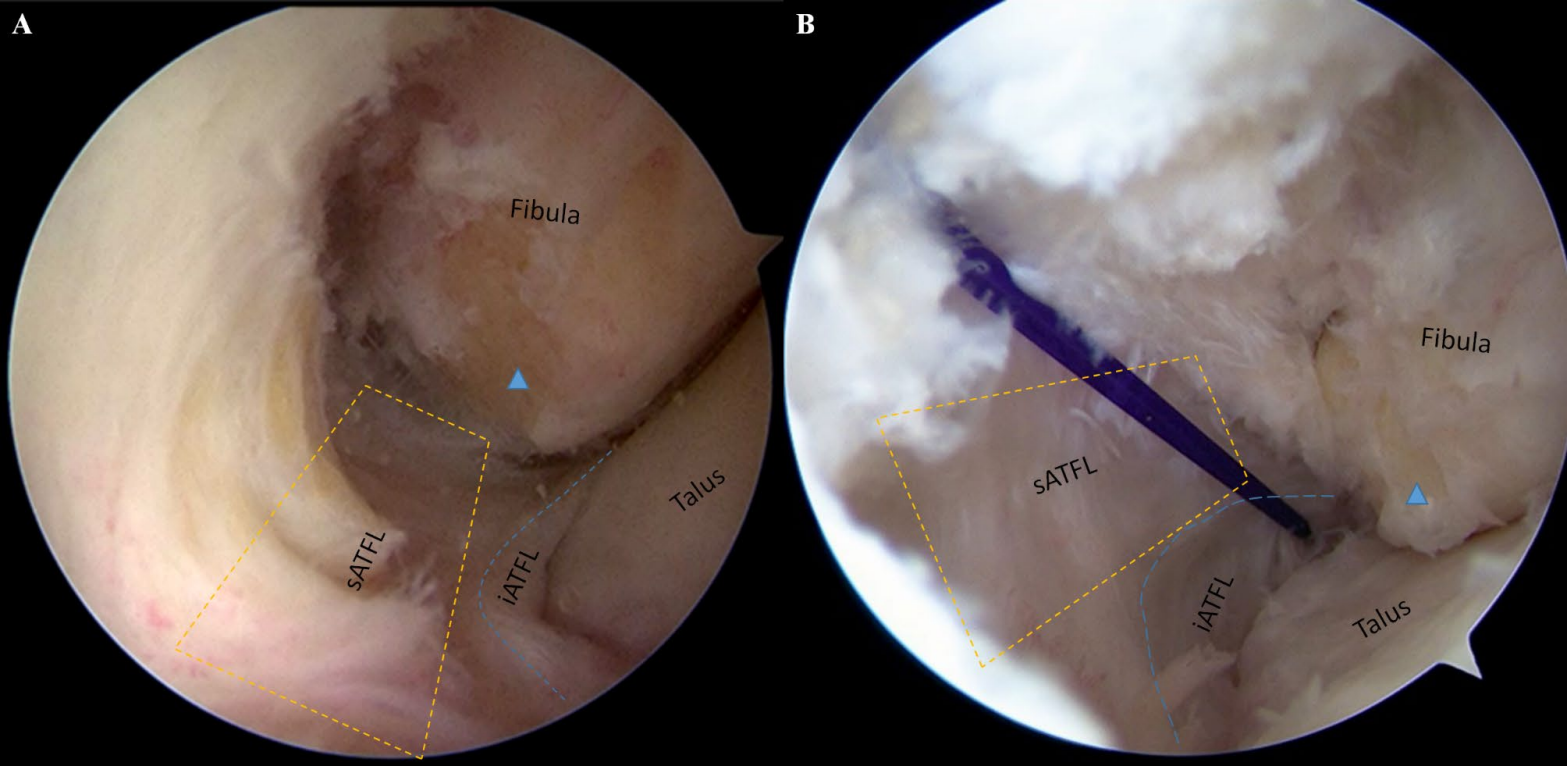
Arthroscopic view of anterior-lateral gutter of ankle joint for anterior-medial portal. After the superior bundle of ATFL (sATFL, orange dotted rectangle) and joint capsule of ankle were released, the inferior bundle of ATFL (iATFL, blue dotted line) could be visualized which was below the LPF (blue solid triangle) and running posteriorly to the fibular tip. to tighten the CFL the suture limb should penetratete out from the deep layer of the iATFL (B, blue-color PDS II suture)

**Fig. 4:**
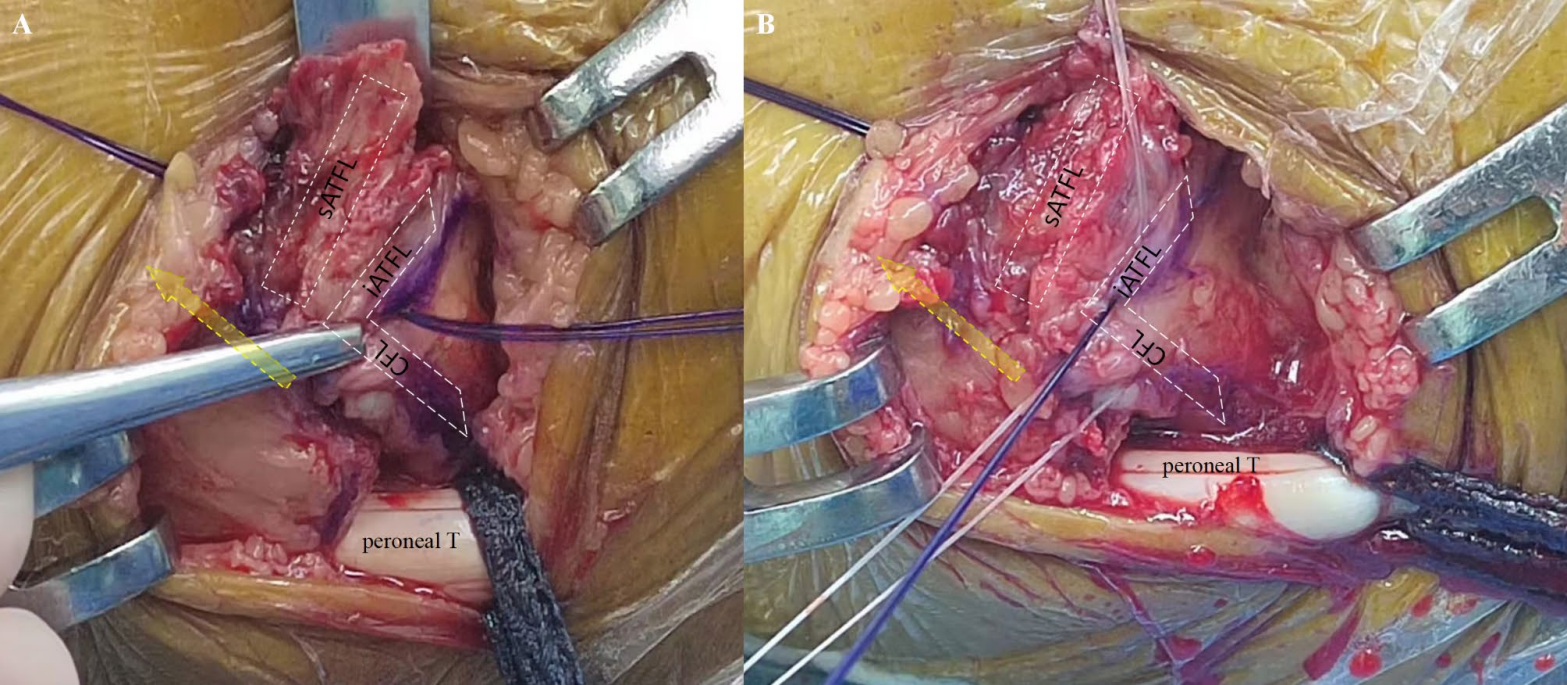
Open procedure was performed in a acute lateral ankle ligament injuried patient with talar insertional rupture of sATFL and fibular insertional rupture of iATFL and CFL. Before excise the inferior extensor retinaculum (IER) and capsule, the PDS II suture was penetrated deep through the iATFL and arciform fiber under arthroscopy. **A**: The penetrating point was located just between the iATFL and CFL and could provide firmly tension to both ligament by one single suture in following open procedure (orange solid arrow: the longitudinal axies of fibule, Peroneal. T: peroneal tendon, sATFL: superior bundle of ATFL, iATFL: inferior bundle of iATFL). **B**: The ruptured ligaments were sutured by three suture anchors (one implanted into talar insertion, and two implanted into fibula insertion)

**Fig. 5:**
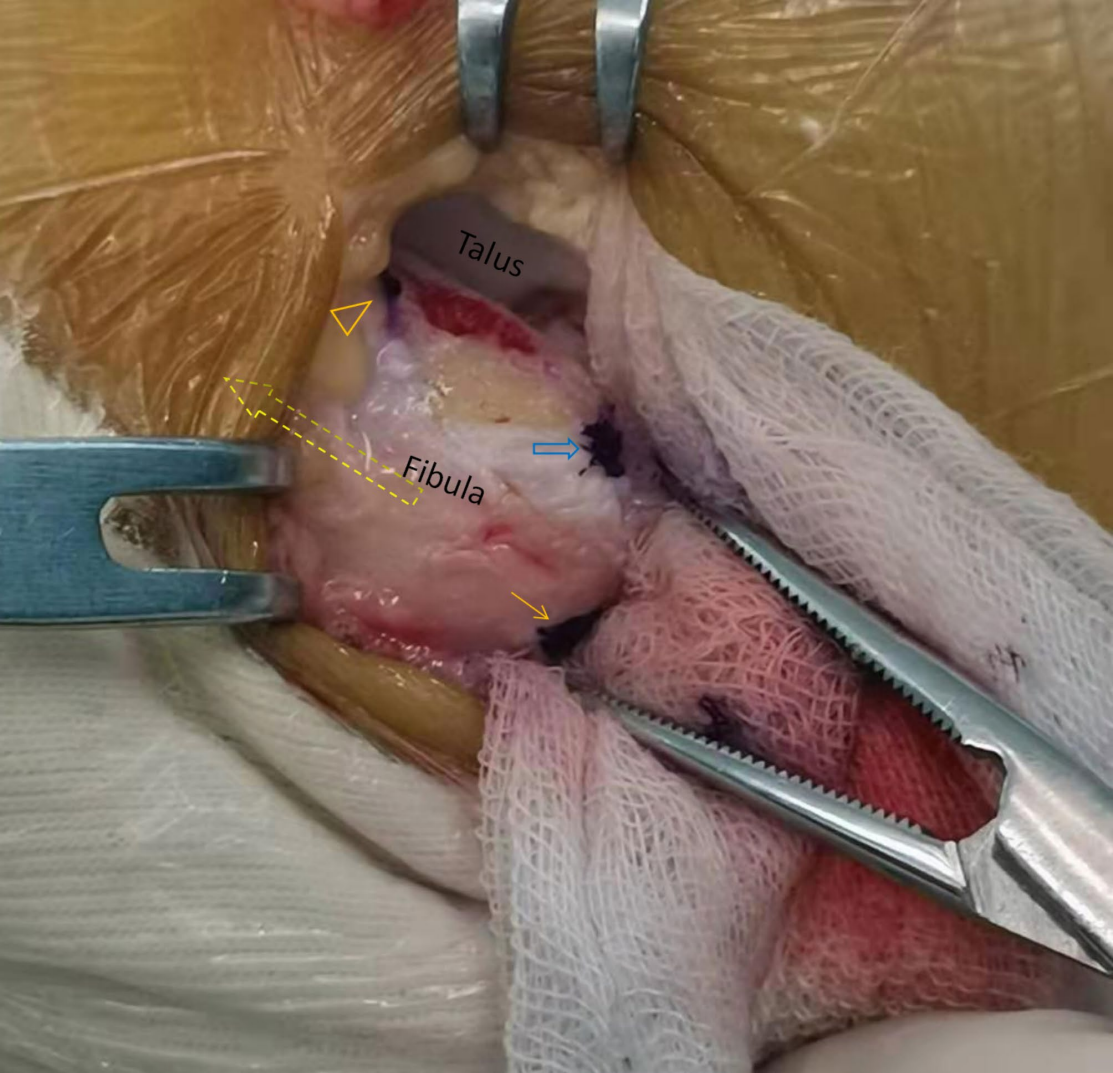
After excision the excise the inferior extensor retinaculum (IER) and lateral ligaments in a chronic lateral ankle ligaments injuried patient, the bony landmarkers of distal fibular were labeled (fibular longitudinal axies: yellow dotted arrow; The fibular insertion of anterior inferior tibiofibular ligament (AITFL): orange solid triangle; FOT: blue empty arrow; Fibular tip: blue solid arrow; Talus: blue *), and the distances of bony landmarks to the bone tunnel were measured

After spinal anesthesia, patients were placed in the semilateral decubitus position, and a pneumatic thigh tourniquet was used to control the pressure at 300 mmHg. Arthroscopic procedure was performed according to the previously study [17], Standard anteromedial (AM) and anterolateral (AL) portals were established, and the intra-articular lesions were investigated and addressed using a 4.0-mm 30^O^ angled arthroscope and a shaver. After the synovial tissue and the fibular insertion of ATFL were debrided, and the lowest point of fibula (LPF) were identified under arthroscopic view via AM portal, and a marker was made at this point by a 4.0-mm radiofrequency ablation. Following that, a 3.0-mm bone tunnel was made at this point by a power drill via AL portal (Fig. 2). The bundle just running inferiorly and posteriorly below the LPF, was identified as iATFL and the arciform fiber (Fig. 3). We labelled this fiber by a blue-color PDS II suture(VICRYL™ Polyglactin 910 Sterile Synthetic Absorbable Surgical Suture PDS™ II (Polydioxanone) Sterile Synthetic Absorbable Surgical Suture ), which was percutaneously introduced by a 16^#^ spinal needle just at the level of the fibula tip, and penetrated through the deep layer of the iATFL, and into the ankle joint under the arthroscopic visualization (Fig. 3B). After that, a traditional open Brostrom procedure was performed [18]. The inferior extensor retinaculum (IER) was dissected and retracted distally, The lateral ankle ligament was exposed and the penetrating point of PDS II suture on iATFL and the arciform fiber was verified (Fig. 4A) intraoperatively, and the distances of penetration point to the horizontal line cross the fibular tip and the vertical line cross the FOT were measured. Following that, the distances of bone tunnel to the fibular tip, the fibular insertion of anterior-inferior tibiofibular ligament (AITFL), and the Fibular obscure tubercle (FOT) were measured using a calliper (Shanghai Measuring and Cutting Tools Company Ltd., Shanghai, China) (Fig. 5);when the measurement completed, the ATFL and CFL were sutured to the fibular insertions using three 2.3-mm suture anchors ( Osteoraptor, Smith and Nephew, Andover, MA, USA) (Fig. 4B). The inferior extensor retinaculum (IER) and capsule were advanced proximal and sutured to the anterior border of the lateral malleolus.

#### Open locating group

The traditional open Brostrom procedure was performed directely, and a 3.0-mm bone tunnel was made at the ATFL insertion referenced to FOT by a power drill after capular dissection. And the measurements and ligaments repair procedure were performed as mentioned above.

The distances of bone tunnel to the fibular tip, the fibular insertion of anterior-inferior tibiofibular ligament (AITFL), and the Fibular obscure tubercle (FOT) were measured and compared between two groups.

Postoperative computed Tomography (CT) evaluation:

A 32-multi-detector-row CT (GE Medical System, Milwaukee, WI, USA) was used in this study with a 0.4 mm slice thickness. Then a 3-dimensional images were reconstructed.

**Fig. 6:**
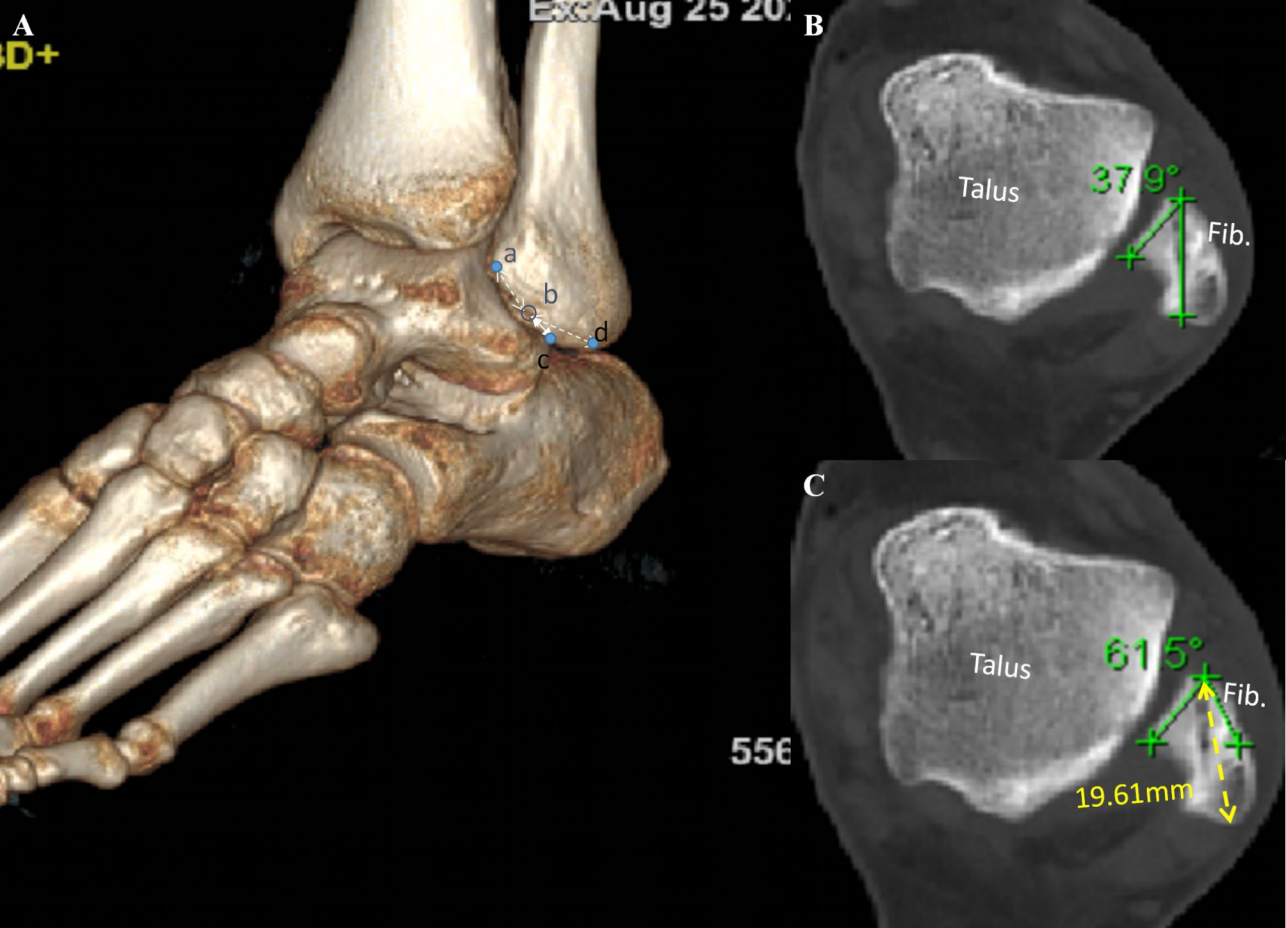
Postoperative 3d-CT scan. **A**: a: anteror fibular tubercle(AFT); b: bone tunnel; c: FOT; c: fibular tip; **B,C**: axial plain of CT scan, fib.: fibula

**Fig. 7:**
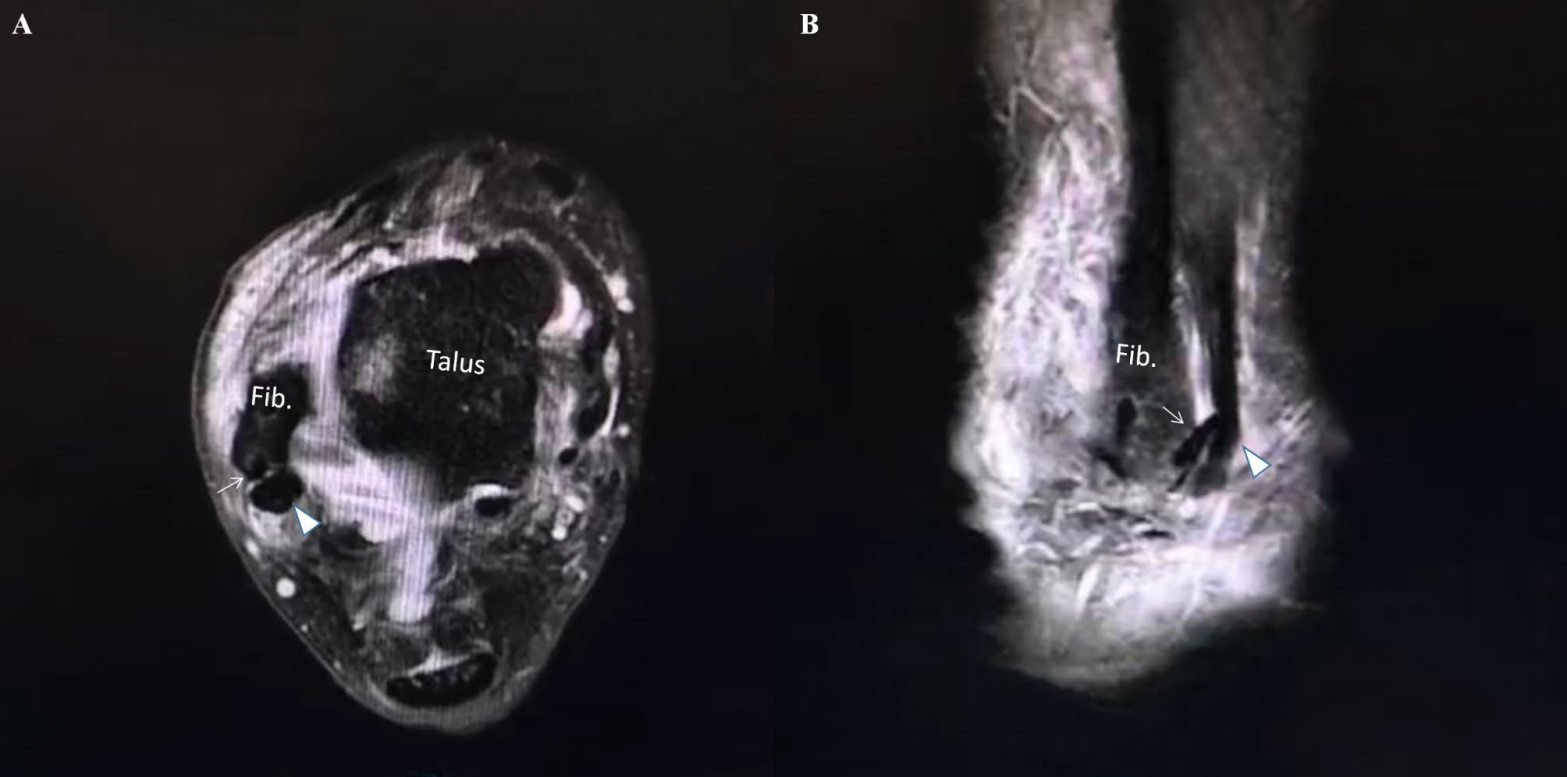
Postoperative MRI in a patient performed a arthroscopic repairing of lateral ankle ligament. **A**: axial view, **B**: sagital view. Fib.: fibule, the suture anchor (white solid arrow) penetrated through the posterior wall of the distal fibule, and disturbed the peroneal tendons (white triangle)

The diameter of bone tunnel which was prepared for suture anchor implantation was calculated as a diameter of 3.0 mm. The distances of bone tunnel to the fibular inferior tip and the FOT were measured on 3D CT oblique sagittal view. The FOT on sagittal view was defined as a corner of the anterior inferior slope of distal fibula from the anterior tubercle of fibula (ATF) to the fibular inferior tip (Fig. 6A) [19]. The 3d-CT measurements were compared between two groups. The angle of suture anchor in axial plane was defined as a angle between the line tangent to articular surface of fibula and the axial line of the drilled bone tunnel (Fig. 6B-C). If the implanted suture anchor was neither penetrated into the fibular articular surface and fibular groove nor broken the lateral wall of bone tunnel, we considered it’s a safe angle in axial plane for suture anchor implantation (Fig. 7).

### Statistical analysis

Statistical analyses were performed using Statistical Package for the Social Sciences (SPSS) version 22.0 (SPSS Inc., Chicago, IL, USA). Means and standard deviations were reported for numerical variables. T test was used to compare between two samples, and p < 0.05 was indicating statistically significant.

## Result

Sixty patients were divided randomly into two groups: arthroscopic locating group (29 cases) and open locating group (31 cases). And the demographic features, including: sex, age, Body Mass Index (BMI), duration of injury, were included and compared between two groups and there were no significant defferences (Table 1).

**Table 1:** Demographic features

Group	Arthroscopic	Open	*P* Value
Number	29	31	
Sex	Male: 17, Female: 12	Male: 17, Female: 14	
Age (Year)	27.6 *± 6.7*	29.5 *± 9.2*	> 0.05
BMI	24.1 *± 2.2*	24.1 *± 3.1*	> 0.05
Duration of injury (Week)	23.3 *± 17.0*	24.1 *± 21.4*	> 0.05

The distances of bone tunnels to FOT was 4.9 ± 2.2 and 4.5 ± 0.7 mm in arthroscopic and open group, respectively (Table 2). The post operative 3d-CT scans were also performed, and the the distances of bone tunnels to the FOT and fibular tip on 3d-CT view was 4.4 ± 1.5 and 4.6 ± 0.9 mm, 14.4 ± 3.2 and 13.2 ± 1.8 mm in arthroscopic and open group, and there were no significant differences between two groups. The safe angle of anchor implantation on axial CT view was from 24.9 ± 6.3^o^ to 58.1 ± 8.0^o^, the anterior-posterior diameter of distal fibular at the FOT was 20.0 ± 2.8 mm. The percutaneous penetration of PDS II suture was also comfirmed after open procedure, and penetrating points of PDS II suture were located on the arciform fiber between iATFL and CFL in all patients (Fig. 4A) (Supplementary 1). The average distance of penetration point to the horizontal line cross the fibular tip was 2.3 ± 2.7 mm (ranged from − 3.1 to 6.0 mm), and to the vertical line cross the FOT was 2.7 ± 2.7 mm (ranged from − 2.5 to 7.5 mm).

**Table 2:** The disatances of bone tunnel to the fibular insertions of AiTFL, FOT, and fibular tip were measured and compared in arthroscopic and open locating groups

Bony landmarkers	FOT	Fibular tip	Fibular insertion of (AITFL)
Arthroscopic group (mm)	4.9 ± 2.2	13.5 ± 2.7	5.8 ± 2.2
Open group (mm)	4.5 ± 0.7	12.4 ± 1.1	5.6 ± 1.0
P value	> 0.05	> 0.05	> 0.05

P>0.05: indicating no significant statistically differences

## Discussion

Arthroscopic repair of lateral ankle ligaments was well accepted by more and more surgeons [17] [18, 20, 21], especially for those injuries of CFL ruptured from fibular insertions. And well understanding the arthroscopic landmarkers of ATFL and CFL footprints and the arthroscopic feature of iATFL and arcute ligaments, were very important for arthroscopic repairing. In traditional anatomic study, AFT (Anterior Fibular Tubercle), FOT, and the fibular tip were the most often used bony landmarkers for suture anchor implantation, and they found the distances of the ATFL to the fibular tip and the FOT was 17.09 ± 5.01 mm, 5.75 ± 2.16 mm, and 6.06 ± 2.58 mm, respectively. distances of ATFL insertion to the FOT was 0.6–15.9 mm and 2.4-6.0 mm (Table 3). But, unlike open procedure condition, it’s very difficulty to visualize the AFT and fibular tip arthroscopically. In present study, we used a landmarker—the lowest point of fibula (LPF) under arthroscopic view as a reference for ligaments identification and suture anchor implantation, and we believed that this landmarker was corresponding to the fibular obscure tubercle(FOT) in open anatomy. The bone tunnels referenced to the LPF in arthroscopic locating group were measured and compared with the results referenced to the FOT in open locating group, the distances of bone tunnels to the fibular tip, the fibular insertion of AITFL, and the FOT was no statistical significance between two groups. Those results were also comparatable to those of previously reported studies (Table 3). And also, in another study, Hattori S et al [15], found that the distances of the anchor and FOT was 6.0 ± 2.7 mm in open repair and 5.6 ± 3.3 mm in sonographically guided repair, it’s very similar to our study, and indicating that the LPF was a very reliable landmarker for suture anchor implantation.

**Table 3:** Distances of the bony landmarkers to the Fibular tip, FOT (Fibular obscure tubercle), and AFT (Anterior Fibular Tubercle), referenced from previously studies

The distance to bony landmarker	Fibular Tip (mm)	FOT (mm)	AFT(mm)	References
ATFL	15.9 ± 3.2	3.7(0–6.7)		[22]
CFL	8.6 ± 2.9	4.9(1.1–10.9)		
ATFL		Intersection of ATFL and CFL : 2.4 (0–6.3)		[11]
CFL				
ATFL	14.3 ± 1.9 (male, 14.7 ± 1.6; female, 13.9 ± 2.0)			[23]
CFL	7.4 ± 1.7 (male, 7.5 ± 1.5; female, 7.3 ± 1.8)			
ATFL		6.0 ± 2.7(open Broström repair); 5.6 ± 3.3(sonographically guided)		[15]
CFL				
ATFL	10.1			[24]
CFL	8.5			
ATFL	10 ± 1.3			[25]
CFL	7.3 ± 1.49			
ATFL	13.32 ± 1.17		20 ± 3.54	[26]
CFL	Just below the ATFL			
ATFL	Single: 13.8; Double: Sup: 16.3, Inf: 10.2;			[27]
CFL	3.5			
ATFL	0.58 ± 1.89		3.45 ± 1.34	[28]
CFL			3.45 ± 1.34	
ATFL	8.4 ± 1.8		16.9 ± 3.1	[29]
CFL	5.0 ± 1.4		21.1 ± 3.1	
ATFL		3.7(0–6.7)		[20]
CFL		4.9(1.1–10.9)		

Cordier G et al [16], carried out a cadaveric study and found out that the iATFL and CFL was connected by the arciform fiber, the this fiber was firm enough to transmit the tension between the iATFL and CFL. In fact, in our cadaveric study (not published), the iATFL, CFL and the arciform fiber constructed a triangle complex, of which the apexs were located at the anterior border of fibular between the fibular tip and FOT, the TOT (Talar Obscure Tubercle) and the calcaneus insertion of CFL, so we could augment the iATFL and CFL by one single limb of the suture anchor under arthroscopy (supplementary 1). Supported by those anatomic studies, we repaired the ATFL and CFL injury with two suture anchors arthroscopically, the one suture anchor was implanted on the lowest point of fibula (LPF) under arthroscopy, and another suture anchor was implanted just above the first suture anchor and below the fibular insertion of AITFL. The key technique point was to identified the iATFL and the arciform fiber under arthroscopy, the suture limbs of the anchor should penetrate deep throught the iATFL and the arciform fiber to tension the CFL and iATFL. the sATFL was repaired using another suture anchor implanted just below the fibular insertion of AITFL.

Nakasa T et al [30], firstly reported the safe angle of the suture anchor implantation on the sagital plain, they concluded that the safe angulations of the anchors implanted direction and the longitudinal axis of fibular should be 34.6 ± 5.0 for ATFL repair, and 15.1 ± 5.7 for CFL repair.In present study, we measured the optimal angle of anchor implantation on axial plain, and difined the safe angle as from 24.92 ± 6.29^o^ to 58.07 ± 8.03^o^ on axial plain, and we also measure the maximum anterior-posterior diameter of distal fibular at the FOT as 19.97 ± 2.82 mm. To avoid the suture anchor’s disturbing articular surface of ankles and the peroneal tendons (Fig. 7), The anchor implantations on FOT should be angulated with the the medial articular surface of distal fibular from 24.92 ± 6.29^o^ to 58.07 ± 8.03^o^ on axial plain, and the longest suture anchor used for lateral ankle ligaments repair should be less than 19 mm. In previous studies [6–8, 31–34], the length of the suture anchors was ranged from 10 to 15 mm, and the 2.3 mm×10 mm suture anchor (Smith&Nephew) was used in our present study, and all of those suture anchors were very suitable for arthroscopic repairing according to our measurements.

## Limitation

There were still remaining a number of limitations in the present study. Firstly, the sample size was not big enough, and because of not a cadaveric study, we couldn’t provide more detailed anatomic data. Secondly, we only presented a bony landmarker—the LPF as a reference to identify the FOT and iATFL under arthroscopy, and did not turely visualized the CFL and its fibula insertion. But those results were sufficient enough to support the possibility of repairing CFL and iATFL injuries arthroscopically. Although it’s a prospective study, we didn’t value the postoperative clinical outcomes of the cases, and didn’t carry out a prospective randomized controlled trials to compare the differences between the clinical outcomes of arthroscopic repairings with or without tightenning the iATFL and CFL.

## Conclusion

Take the lowest point of fibula under arthroscopy (LPF) as a bony reference, we could identify the iATFL under arthroscopic visualization. By this way, we could place the suture anchors properly to the fibular footprint and suture the iATFL fibres successfully.

### Electronic supplementary material

Below is the link to the electronic supplementary material.

Supplementary Material 1

## Data Availability

The datasets of the current study are available from the corresponding author upon reasonable request. The data that support the findings of this study are available on request from the corresponding author.
